# Cervical precancerous and cancerous lesions screening using Pap smear test at Provincial Referral Hospital of Bukavu, Eastern DR Congo: profile and recommendations to stakeholders

**DOI:** 10.11604/pamj.2024.47.57.39090

**Published:** 2024-02-08

**Authors:** Daniel Garhalangwanamuntu Mayeri, Pierre Mulumeoderhwa Kahasha, Isaac Barhishindi Kibalama, Jules Mongane, Medina Louguè, Etienne Kajibwami Birindwa, Serge Chentwali Mwimangire, Claude Kalegamire Kikuru, Jeanne Maningo Materanya, Yvette Kujirakwinja Bisimwa, Benjamin Kasago, Léon-Emmanuel Mubenga Mukengeshai

**Affiliations:** 1 Department of Pathology, Faculty of Medicine, Hôpital Provincial Général de Référence de Bukavu, Université Catholique de Bukavu, Bukavu, Democratic Republic of Congo,; 2 Ecole Régionale de Santé Publique, Université Catholique de Bukavu, Bukavu, Democratic Republic of Congo,; 3 Department of Pathology, Université Joseph Ki-Zerbo, Ouagadougou, Burkina Faso,; 4 Department of Gynaecology, Faculty of Medicine, Hôpital Provincial Général de Référence de Bukavu, Université Catholique de Bukavu, Bukavu, Democratic Republic of Congo,; 5 Department of Surgery, Faculty of Medicine, Hôpital Provincial Général de Référence de Bukavu, Université Catholique de Bukavu, Bukavu, Democratic Republic of Congo

**Keywords:** Cervical cancer, Pap test, DR Congo

## Abstract

**Introduction:**

cervical cancer is a health concern worldwide. The South Kivu Province in the Eastern DR Congo is facing many cases of this disease but poorly screened and reported. The objective of this was to determine the prevalence of cell abnormalities at cervical cytology in a tertiary teaching hospital in Bukavu and their association with common risk factors of cervical cancer.

**Methods:**

a cross-sectional study was conducted on 142 women attending the Provincial Referral Hospital of Bukavu (HPGRB) from February to December 2021. Quantitative variables were described by their median following their asymmetric distributions and the qualitative variables in absolute and relative frequencies. Then the Chi-square test was used for the comparison of proportion.

**Results:**

forty-five percent of the participants had between three and five children. Twenty-two (15.5%) of the 142 patients reported to have two or more sexual partners and 17.5% reported the use of hormonal contraception. The prevalence of cell abnormalities at cervical cytology was 17% of which Low- Grade Squamous Intraepithelial Lesion (LSIL) was the most representative (12.9%). There was no statistically significant association between the common cervical risk factors and the occurrence of cell abnormalities.

**Conclusion:**

cervical pre-cancerous lesions are frequent in South Kivu province. The Pap smear test remains an early and affordable screening method and constitutes a secondary prevention strategy in women of 18 years and older in a low-income country such as DR Congo where vaccination against HPV is still hypothetic.

## Introduction

Cervical cancer is a health concern worldwide. About 604,000 new cases of uterine cervical cancer were reported in 2020 in the world of which 342,000 persons died of this disease [1]. Eighty to eighty-five percent of these cervical cancer cases occur in low-income countries [2,3]. This is due to the fact that in developing countries, patients go to health facilities late and the diagnosis is made at an advanced stage. However, in high-income countries such as the United States of America and Europe, the incidence rate is about 6 to 10 per 100,000 women per year [2,3] and the number of deaths due to cervical cancer is 10 times less high than in low-income countries. The difference is explained by the occurrence of the major risk factors at the time of screening. Human Papillomavirus infection has been identified as the most important risk factor. Many other risk factors have been found to be prominent in low-income countries such as early age of first sexual intercourse, many lifetime sexual partners [4], the number of childbirths, the long term hormonal contraception [5,6] and tobacco smoking [7].

In Africa, the cervical cancer is the second cause of death in women with an incidence rate of 25 per 100,000 women per year and is increased up to 35 per 100,000 women per year in sub-Saharan Africa [8]. In 2020, WHO using GLOBOCAN 2020 has estimated that the incidence of cervical cancer has increased especially in East Africa where as incidence became stable at relatively low levels around 2005 in several high-income countries. The reason is the fact that some of these countries such Uganda have unsufficient and opportunistic services [9].

In the other side, the cervical cytology by Pap smear test is one of the current available and recommended methods in cervical cancer screening. Other methods include liquid-based cytology; HPV DNA testing using the conventional PCR method or hybrid capture; and biomarkers of molecular pathways in cervical carcinogenesis, such as early proteins (E6 and E7) mRNA detection and p16 cell-cycle protein levels. Among all these screening methods, the Pap smear test has the advantage to be the oldest but also the most effective and cheapest screening methods of cervical cancer in low-income countries [10]. Although the conventional smear test is in the process of being replaced by the HPV test, it still has some advantages. It is inexpensive and acceptable [11]. In Democratic Republic of the Congo, we know next to nothing about this concern.

Some studies conducted in Kinshasa in the west of the country have found that the prevalence of precancerous lesions among women in different neighborhoods of Kinshasa is about 4% with HPV infection and HIV infection as associated risk factors [12]. In eastern DR Congo, where people are facing high rate of HIV infection and unwanted pregnancies at a young age witnessing an early sexual intercourse probably because of the frequent armed-conflicts in region, very few studies have been conducted. Paluku *et al*. in Goma, the capital city of North Kivu Province, near Bukavu city, has conducted a study on massive single visit cervical pre-cancer and cancer screening. His findings have highlighted that the prevalence of precancerous lesions was 2.34% and the prevalence of squamous cell carcinoma 0.93% [13]. Recently, a study conducted at Panzi Hospital in South Kivu Province has assessed the sensitivity and predictive value of the HPV test associated with uterine cervical-smear in screening for intraepithelial neoplasia of the cervix in our environment [14]. In their study, the prevalence of cell abnormalities was 14.7% versus 82.8% of normal cervical uterine smear. This study has then focused on the sensitivity of HPV test but did not deeply explore the role of the Pap smear test in the detection of cervical precancerous lesions.

Moreover, they did not assess the association of common cervical cancerous risk factors and the observed cell abnormalities. Considering this particular situation of the Eastern DR Congo where there is no organized screening cervical cancer in addition to the existence of sexual abuse due to armed conflicts and the previously described health concerns, studies focusing on cervical cytology and risk factors of cervical precancerous lesions especially in South Kivu province are scarce. This study aimed to describe the cervical cytology profile of patients attending the Provincial referral hospital of Bukavu. Specifically, this study aims to determine the prevalence of cell abnormalities at cervical cytology in a tertiary teaching Hospital in Bukavu and their association with common risk factors of cervical cancer. Since this tertiary hospital receives patients from South and neighbors from North-Kivu and Western Rwanda and Burundi, two border countries, this study constitutes a baseline study for future large-scale studies in order to enhance and promote cervical cancer screening policies and will then allow to call for more preventive actions by stakeholders.

## Methods

**Study design setting and participants:** from February to July 2021, we conducted a cross-sectional study on cervical precancerous and cancerous lesions screening. A total of 142 women older than 17 years who attended the Department of Gynecology of the Provincial Referral Hospital of Bukavu and who freely accepted a Pap smear test were screened. Exclusion criteria were pregnancy, severe gynecological bleeding, and previous hysterectomy and age <18 years.

**Data collection and variables settings:** before proceeding to sample collection, participants were asked about some common risk factors of the cervical cancer including the age the first sexual intercourse, the number of sexual partners, the number of pregnancies and the use of hormonal contraception. Then the Pap smear samples were collected by gynecologists and directly sent to the Department of Pathology for Papanicolaou test [15] stain after what two trained pathologists read the slides independently. On cytology, a Papanicolaou smear test was set according to the 2014 Bethesda System [16]. Women with Cell abnormalities including atypical squamous cells of undetermined significance (ASCUS), atypical squamous cells, cannot rule out high-grade lesion (ASC-H), Low- Grade Squamous Intraepithelial Lesion (LSIL) and High-Grade Intraepithelial Lesion (HSIL) were considered to have precancerous and cancerous lesions (HSIL/carcinoma in situ) while women with NILM (negative for intraepithelial lesion and malignancy) or inflammation were considered to be free of cancer.

**Ethics approval and consent to participate:** the protocol of this this study received approval by the Catholic University of Bukavu Ethics committee under approval number UCB/CIES/02-07/2020. Informed consent was obtained from all subjects and all methods were performed in accordance with the relevant guidelines and regulations (Declaration of Helsinki) [17].

**Data analysis:** we used the Excel program for data entry. The data were then exported to Epi info 7 and SPSS 25 for processing and analysis. The quantitative variables were described by their median following their asymmetric distributions and the qualitative variables in absolute and relative frequencies. The Chi-square test was used for the comparison of proportions.

## Results

**Socio-demographic and behavior-related characteristics of the patients:** a total of 142 patients were included in this study. The participant's age ranged from 18 and 80 years old with the median age of 37.5 years. More than 56% got their first sexual intercourse before the age of 16 years. Forty-five percent of the participants had between three and five children (Table 1). Twenty-two (15.5%) of the 142 patients reported to have two or more sexual partners and 17.5% reported the use of hormonal contraception (Table 2).

**Table 1:** socio-demographic characteristics of the study population

	N	%
**Age of patients (years) N=142**		
Median (Min-Max)	**37.5 (18-80)**	
**Age of first sexual intercourse (years)**		
Median (Min-Max)	**15** (12-18)	
≤15 years	**98**	**69.0**
≥16 years	**44**	**31.0**
**Employment N=142**		
State employees	**10**	**7.0**
Students	**18**	**12.7**
Businesswoman	**13**	**9.1**
Household	**89**	**62.7**
None	**12**	**8.5**
**Number of pregnancies N=142**		
0-5	**98**	**69.0**
≥6	**44**	**31.0**
**Number childbirths**		
0-5	**106**	**74.6**
≥6	**36**	**25.4**

**Table 2:** behavior-related characteristics of the study population

	n	%
**Number of sexual partners**		
01	**120**	**84.5**
≥2	**22**	**15.5**
**Use of hormonal contraception**		
Yes	**25**	**17.6**
No	**117**	**82.4**

**Cytology results in light of the 2014 Bethesda System:** more than the half of the study population had a non-neoplastic cervicitis (56.1%) of which non-specific inflammation was prominent. The prevalence of cell abnormalities at cervical cytology was 17% of which Low- Grade Squamous Intraepithelial Lesion (LSIL) was the most representative (12.9%) (Table 3).

**Table 3:** cytology results in light of the 2014 Bethesda system

	N	%
**Normal cytology**	**38**	**26.8**
**Non-neoplastic cervicitis(n=80)**		
Candida ssp	**4**	**2,8**
Nonspecific inflammation	**76**	**53,3**
**Epithelial Cell abnormalities (24)**		
**ASCUS**	**3**	**2.1**
**LSIL**	**18**	**12.9**
**HSIL**	**3**	**2.1**
**Total**	**142**	**100**

**Bivariate associations between socio-demographic and behavioral characteristics and presence of cell abnormalities at Pap smear test:** the Table 4 summarizes the results of the bivariate association between socio-demographic, behavioral characteristics and presence of cell abnormalities. Almost 5% of women who had their first sexual intercourse at age≥16 years, 8% of those who have had ≥6 pregnancies, 4.2% of women who have had two or more sexual partners and 3% of those who have used hormonal contraception have developed cell abnormalities. But this association was not statistically significant.

**Table 4:** bivariate associations between socio-demographic and behavioral characteristics and presence of cell abnormalities at Pap smear test

	All (N=142)	Normal (n= 118)	Cell abnormalities (n=24)	P Value
**Age of first sexual intercourse**		N (%)	N (%)	0.833
≤15 years	-	81 (57)	17 (12)	
≥16 years	-	37 (26.1)	7 (4.9)	
**Number of pregnancies**	-	N (%)	N (%)	0.084
0-5	-	85 (60)	13 (9)	
≥6	-	33 (23)	11 (8)	
**Number of sexual partners**	-	N (%)	N (%)	0.158
0-1	-	102 (72)	18 (12.6)	
≥ 2	-	16 (11.2)	6 (4.2)	
**Use of hormonal contraception**	-	N (%)	N (%)	0.895
Yes	-	21 (14.7)	4 (3)	
No	-	97 (68.3)	20 (14)	

## Discussion

This study described the profile of the cervical cytology of women attending a provincial hospital in Bukavu using the Pap smear test. It performed the assessment of the prevalence and risk factors of precancerous lesions. To our knowledge, this health concern has not been addressed as such with the same settings before in South Kivu Province, a conflict affected province in the Eastern DR Congo. The overall characteristics of this study are consistent with the literature [12,13].

The prevalence of cell abnormalities at cervical cytology in this study was 17% of which Low- Grade Squamous Intraepithelial Lesion (LSIL) was the most representative (12.9%). This prevalence is the highest of the few previous findings by Hoyland *et al*., 2010 and Nyakio *et al*., 2021) in Eastern Democratic Republic of Congo which was 7% and 14.7% in Bukavu city in the South Kivu province [14,18] and 2.3% in Goma city by Paluku *et al*., 2019 in the North Kivu Province [13]. Moreover, this prevalence is higher than the reported by Laga *et al*.,1992; Ali-Risasi *et al*., 2015 and Sangwa-L *et al*., 2006 in Kinshasa, the capital city of the DR Congo which varies from 3% to 5% [19,20]. This highest prevalence may be explained by the fact that the South Kivu Province has been facing armed conflict and sexual violence for decades and up to know this dramatic situation is yet to end. These facts and their association to cervical cancers need to be assessed by future studies.

The difference is also observed elsewhere in Sub-Saharan Africa where lower prevalence than the one found in this study has been reported and varied from 4% to 10%. These countries include Burkina Faso [21], Nigeria [22,23], Kenya [24]. These differences may also be explained by the unavailability of the HPV vaccine in DRC despite the efforts of the Gavi vaccine alliance that aimed to vaccinate 30 millions of girls in Africa [13]. There was an association between the age of first sexual intercourse, the number of pregnancies, the number of sexual partners, the use of hormonal contraception and the occurrence of cell abnormalities but this was not statistically significant. This is consistent with findings in Kinshasa by Ali-Risasi *et al*., 2015 [20] who did not find a statistically significant association between having three or more sexual partners and the occurrence of cell abnormalities among the study population. This may be explained by the small sample size in our study and calls for a large scale study in order to assess the relationship between these common risk factor and the occurrence of cervical cancer in the context of South Kivu Province. However, her findings has showed an association between the use of plants for vaginal care and the occurrence of precancerous lesions. In our study, we did not, however, assess this aspect. Since cultural behavior in Kinshasa and Bukavu in vaginal hygiene is almost similar, this aspect needs to be assessed in future studies.

**Strengths and weakness of the study:** this study has described the profile of cervical cytology using Pap smear test in a cervical precancerous screening purpose. It has estimated the prevalence of these lesions and the possible association with some known risk factors in a tertiary hospital that deserves almost the Eastern DR Congo. To our knowledge, this is the first time that such a profile is addressed in an armed-conflict affected province such as South Kivu using a simple, affordable and acceptable screening method.

However, this study has some limitations. The cross-sectional design of this study does not allow us to establish a cause-effect relationship between risk factors and cancerous lesions. Moreover, since this study has used hospital data, this may have caused selection bias. The small sample size may have impacted negatively on the prevalence of cell abnormalities. The Pap smear used to collect cytology data has been questioned by scientists because of the need for repetitive smears (due to the low sensitivity), the recall of patients for their results and the need for a laboratory with high human expertise.

Despite all this, this study constitutes a baseline for future studies. Thus, prospective studies addressing the prevalence and risk factors of cancerous lesions as well as those addressing attitudes and knowledge of women about cervical cancer are needed.

## Conclusion

Cervical pre-cancerous lesions are frequent in South Kivu province. The Pap smear test remains an early and recommended and affordable screening method in women of 18 years and older in a low-income country such as DRC. Prevention actions based on a systematic Pap smear test in this category are encouraged since vaccination against HPV is still hypothetic in DRC.

### What is known about this topic

- *Some studies conducted in Eastern DRC in Goma and Bukavu has showed a high prevalence (2-14%) of cervical pre-cancerous and cancerous lesions;*
- *These studies have used Pap smear test combined Pap smear test and molecular biology;*
- *In Goma, the “screen and treat” approach was used; these methods are sensitive but not affordable buy the people in such a region whose daily income is less than USD 2*.

### What this study adds

- *Although the small sample size and the fact that data were collected in a tertiary hospital, this study shows that precancerous lesions are still prevalent despite all prevention strategies undertaken by stakeholders against cervical cancer;*
- *The prevalence found in this study is higher than that found previously in the same region (17%); moreover, this study has used Pap smear test itself which an affordable screening method in a context of a low income country;*
- *This study reinforces previous recommendations on this topic and highlights the need of vaccination against HPV in DR Congo in order to reduce the prevalence of cervical cancer*.
